# Efficacy and safety of finerenone in obesity-related glomerulopathy

**DOI:** 10.1093/ckj/sfaf157

**Published:** 2025-06-04

**Authors:** Dan-dan Qiu, Jing Liu, Rui-han Chen, Feng Zhang, Yu An, Song Jiang

**Affiliations:** National Clinical Research Center for Kidney Diseases, Jinling Hospital, Affiliated Hospital of Medical School, Nanjing University, Nanjing, China; National Clinical Research Center for Kidney Diseases, Jinling Hospital, Affiliated Hospital of Medical School, Nanjing University, Nanjing, China; National Clinical Research Center for Kidney Diseases, Jinling Clinical Medical College, Nanjing University of Chinese Medicine, Nanjing, China; National Clinical Research Center for Kidney Diseases, Jinling Hospital, Affiliated Hospital of Medical School, Nanjing University, Nanjing, China; National Clinical Research Center for Kidney Diseases, Jinling Hospital, Affiliated Hospital of Medical School, Nanjing University, Nanjing, China; National Clinical Research Center for Kidney Diseases, Jinling Hospital, Affiliated Hospital of Medical School, Nanjing University, Nanjing, China

**Keywords:** efficacy, finerenone, obesity-related glomerulopathy, safety

## Abstract

**Background:**

This study aims to evaluate the efficacy and safety of finerenone in the treatment of obesity-related glomerulopathy (ORG).

**Methods:**

A retrospective analysis was conducted on 69 patients diagnosed with ORG between January 2022 and July 2023, of whom 30 received finerenone (10–20 mg/day).

**Results:**

The cohort had a mean age of 44.30 ± 11.43 years, comprising 54 males. The median body mass index (BMI) was 31.18 (28.89, 33.68) kg/m², the median 24-hour proteinuria level was 1.35 (1.2, 1.86) g/24 h, the mean estimated glomerular filtration rate (eGFR) was 87.39 ± 28.41 ml/min/1.73 m², and the mean serum potassium level was 4.01 ± 0.33 mmol/l. All patients were followed for over 1 year. Compared to the control group, the finerenone group had a lower baseline BMI [29.86 (28.66, 32.91) vs. 31.67 (30.18, 34.56) kg/m², *P* = .019] and higher baseline proteinuria [1.72 (1.23, 2.63) vs. 1.32 (1.12, 1.66) g/24 h, *P* = .007]. The utilization of renin–angiotensin system (RAS) inhibitors, sodium-glucose cotransporter-2 (SGLT2) inhibitors, glucagon-like peptide-1 (GLP-1) receptor agonists, and statins showed no significant differences between the groups. At 1-year follow-up, the finerenone group demonstrated significantly greater reduction in 24-hour proteinuria (−35.03% vs. −11.20%, *P* = .010) and systolic blood pressure (−10.07 vs. −4.44 mmHg, *P* = .045), along with a more stable eGFR (2.85% vs. -8.20%, *P* = .009) compared with the control group. Additionally, serum potassium levels increased more in the finerenone group (8.09% vs. 1.73%, *P* = .005). No significant difference in adverse events were observed between the groups.

**Conclusions:**

Finerenone is associated with reduced proteinuria, lower blood pressure, and stabilized eGFR in patients with ORG, without a significant increase in adverse events.

## Graphical Abstract

**Graphical Abstract:**
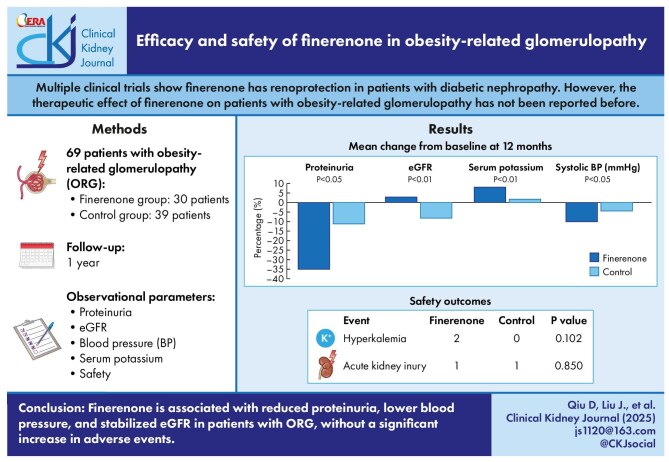

KEY LEARNING POINTS **What was known**:

- Multiple clinical trials have shown that finerenone significantly reduces the urine albumin-to-creatinine ratio and slows down the deterioration of kidney function in patients with type 2 diabetes mellitus and chronic kidney disease.
- Obesity-related glomerulopathy (ORG) is another common metabolic kidney disease. However, the therapeutic effect of finerenone on patients with ORG has not been previously reported.

**This study adds**:

- Finerenone use shows correlation with reduced urinary protein levels, lower blood pressure, and attenuated kidney function decline in ORG patients already on maximum tolerated doses of renin–angiotensin system inhibitors.
- The addition of finerenone does not increase the incidence of hyperkalemia and acute kidney injury in patients with ORG.

**Potential impact**:

- Our findings highlight that combining finerenone with the maximum tolerated doses of renin–angiotensin system inhibitors can provide additional renal benefits.

## INTRODUCTION

Obesity-related glomerulopathy (ORG) is a prevalent complication associated with obesity, with recent reports indicating a 10-fold increase in its incidence [1]. Data from our center reveal that the proportion of renal biopsies exhibiting ORG increased from 0.63% during the 1972–2002 period to 4.94% during the 2003–2014 period, highlighting a significant upward trend [2]. According to epidemiological studies, patients diagnosed with ORG may progress to chronic kidney disease (CKD), with ∼10%–30% ultimately developing end-stage kidney disease (ESKD) [3]. Therefore, the identification of effective therapeutic strategies to mitigate the progression of kidney function in ORG is of paramount importance.

The activation of the renin–angiotensin system (RAS) is an integral part of the pathophysiological mechanisms underlying ORG [4]. Currently, RAS inhibitors are widely considered the standard therapeutic agents in the treatment of ORG. Additionally, sodium-glucose cotransporter 2 (SGLT2) inhibitors and glucagon-like peptide-1 (GLP-1) receptor agonists are recommended for treating obesity and its related complications due to their dual effects on weight loss and renoprotection [5]. Furthermore, adipose tissue can secrete aldosterone-releasing factors that stimulate aldosterone secretion independently of the systemic RAS, contributing to the hyperaldosteronism and increased salt sensitivity of blood pressure commonly observed in obese patients [6]. Consequently, the aldosterone/mineralocorticoid receptor system is emerging as a promising therapeutic target for ORG.

Finerenone is a novel, non-steroidal selective mineralocorticoid receptor antagonist (MRA) that offers enhanced anti-inflammatory and anti-fibrotic effects compared to traditional steroidal MRAs, while also exhibiting a reduced incidence of hyperkalemia and deterioration of kidney function. This improvement is attributed to its greater selectivity and affinity for the receptor [7, 8]. Clinical trials have demonstrated that finerenone effectively reduces the urine albumin-to-creatinine ratio (UACR) in patients with diabetic nephropathy (DN) [9]. In individuals with type 2 diabetes mellitus (T2DM) and CKD, the addition of finerenone has been shown to slow the progression of CKD and decrease the incidence of cardiovascular events [10, 11]. However, the therapeutic effects of finerenone in patients with ORG have yet to be reported.

This study employed a retrospective cohort of Chinese ORG patients to investigate the effects of finerenone on proteinuria, blood pressure, and eGFR. The findings indicate that finerenone treatment (10–20 mg/day) significantly reduces proteinuria and blood pressure levels while slowing eGFR decline.

## MATERIALS AND METHODS

### Patients

**Figure 1:**
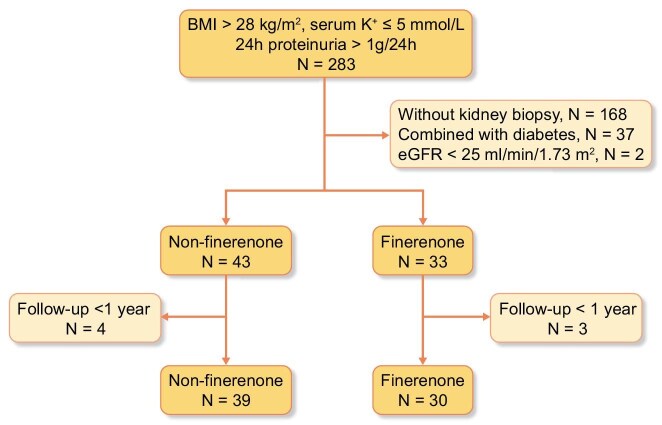
The flowchart of patients screening.

This retrospective analysis included 69 patients diagnosed with ORG through renal biopsy at the National Clinical Research Center for Kidney Diseases between January 2022 and July 2023. The inclusion criteria were as follows: (i) age >18 years; (ii) obesity [baseline body mass index (BMI) >28 kg/m^2^] with biopsy-proven ORG meeting all following [12]: (a) histopathological evidence of glomerulomegaly (glomerular diameter >200 μm); (b) with or without secondary focal segmental glomerulosclerosis (FSGS) (predominantly perihilar variant); (c) exclusion of diabetic nephropathy, hypertensive nephropathy, and primary FSGS by clinical-pathological correlation; (iii) baseline 24-hour proteinuria >1 g/24 h; and (iv) baseline serum potassium ≤5 mmol/l. The exclusion criteria included: (i) a confirmed diagnosis of diabetes mellitus; (ii) concomitant renal diseases as indicated by kidney biopsy; (iii) baseline estimated glomerular filtration rate (eGFR) <25 ml/min/1.73 m^2^; and (iv) a follow-up duration of <1 year. The screening process is illustrated in Fig. 1. Among the 69 patients, 30 were treated with finerenone (10–20 mg) and were classified as the finerenone group, while the remaining 39 patients who did not receive finerenone constituted the control group.

This study was approved by the Ethics Committee of the Jinling Hospital (2022DZKY-069–01) and adhered to the principles of the Declaration of Helsinki. Finerenone was not approved for non-diabetic CKD in China during the period of 2022–2023. All participants provided informed consent, which explicitly covered the use of novel therapies and the collection of samples. Since the initial informed consent addressed the potential use of finerenone, no additional consent was required.

### Data collection and observational parameters

Clinical and laboratory data collected within 1 month prior to the initiation of treatment served as baseline data for the finerenone group. For the control group, baseline data were gathered within 1 month before and after renal biopsy. The baseline data included age, sex, BMI, blood pressure, 24-hour proteinuria, serum creatinine, eGFR, serum potassium, total cholesterol, triglycerides, glycated hemoglobin, and treatment regimens. Follow-up data were collected at 6 months and 1 year, including blood pressure, 24-hour proteinuria, serum creatinine, eGFR, serum potassium, and the incidence of complications.

The observational parameters included the trends in 24-hour proteiniuria, eGFR, blood pressure, and serum potassium levels at 6 months and 1 year after treatment, as well as the incidence of adverse events.

### Treatment regimen

Both groups received the maximum tolerated dose of RAS inhibitors, including ACE (angiotension converting enzyme) inhibitors (ACEIs) and angiotensin-receptor blockers (ARBs), provided that their blood pressure remained within an acceptable range. The treatment regimen for the control group included either monotherapy with RAS inhibitors or combination therapy with metformin, sodium-glucose cotransporter 2 inhibitors (SGLT2i), glucagon-like peptide-1 receptor (GLP-1R) agonists, or statins. In the finerenone group, finerenone was administered at a dose of 10–20 mg/day and adjusted according to eGFR levels, in addition to the RAS inhibitors, metformin, SGLT2i, GLP-1R agonists, or statins.

### Statistical analysis

Statistical analysis was performed using IBM SPSS version 21.0 software. The ratio of 24-hour proteinuria and eGFR was calculated as the follow-up value divided by the baseline value. The percentage changes in 24-hour proteinuria, eGFR, and blood pressure were calculated using the formula: (follow-up value − baseline value)/baseline value × 100%. Continuous variables are presented as the mean ± standard deviation (SD) for normally distributed data, or as median and interquartile range (IQR) for skewed data. Categorical variables are presented as absolute numbers along with their corresponding rates. For intergroup comparisons of continuous variables with a normal distribution, the independent samples *t*-test was utilized. For non-normally distributed continuous variables, the Mann–Whitney *U*-test was employed. The Chi-squared test or Fisher's exact test was applied for the comparison of categorical variables. All tests were two-sided, with a *P* value of <.05 considered statistically significant.

## RESULTS

### Baseline characteristics

A total of 69 patients with ORG were enrolled in the study, including 30 patients in the finerenone group and 39 patients in the control group. The mean age of these patients was 44.30 ± 11.43 years, with 54 males and 15 females. The median BMI was 31.18 (28.89, 33.68) kg/m², and the median 24-hour proteinuria was 1.35 (1.2, 1.86) g/24 h. The mean eGFR was 87.39 ± 28.41 ml/min/1.73m², while the mean serum potassium level was 4.01 ± 0.33 mmol/l.

When compared to the control group, patients in the finerenone group had a lower baseline BMI [29.86 (28.66, 32.91) vs. 31.67 (30.18, 34.56) kg/m^2^; *P* = .019] and a higher baseline 24-hour proteinuria [1.72 (1.23, 2.63) vs. 1.32 (1.12, 1.66) g/24 h; *P* = .007]. Additionally, there were five patients in the finerenone group with 24-hour proteinuria exceeding 3.5 g/24 h, compared to only one patient in the control group. No statistically significant differences were noted between the two groups regarding baseline age, sex, blood pressure, eGFR, serum lipids, glycated hemoglobin, and serum potassium levels. Specifically, the distribution of eGFR, the proportion of patients with an eGFR ≥60 ml/min/1.73 m^2^ was 80% in the finerenone group and 87.2% in the control group (Table 1).

**Table 1:** Demographic and clinical characteristics of the patients at baseline.

Characteristics	Control (*n* = 39)	Finerenone (*n* = 30)	*P* value
Age, year	43.31 ± 10.87	45.60 ± 12.18	.413
Sex, male (%)	32 (82.1%)	22 (73.3%)	.384
BMI, kg/m^2^	31.67 (30.18, 34.56)	29.86 (28.66,32.91)	.019
SBP, mmHg	130.8 ± 14.3	137.2 ± 12.7	.056
DBP, mmHg	80.2 ± 15.7	86.27 ± 12.5	.088
Diagnosed with hypertension, *n* (%)	32 (82.1%)	23 (76.7%)	.812
24 hours proteinuria, g/24h			
Median (IQR)	1.32(1.12, 1.66)	1.72 (1.23, 2.63)	.007
Distribution, *n* (%)			
1–3.5 g/24 h	38 (97.4%)	25 (83.3%)	
>3.5 g/24 h	1 (2.6%)	5 (16.7%)	
Serum creatinine, mg/dl	0.99 (0.74,1.26)	1.10 (0.90,1.33)	.162
eGFR, ml/min/1.73 m^2^			
Mean	92.38 ± 26.53	80.90 ± 29.9	.096
Distribution, *n* (%)			
≥90 ml/min/1.73 m^2^	20 (51.3%)	9 (30.0%)	
≥60 ml/min/1.73 m^2^	14 (35.9%)	15 (50.0%)	
45–60 ml/min/1.73 m^2^	3 (35.9%)	4 (13.3%)	
25–45 ml/min/1.73 m^2^	2 (5.1%)	2 (6.7%)	
Total cholesterol, mmol/l	4.70 ± 0.87	4.72 ± 1.23	.943
Total triglycerides, mmol/l	2.21 (1.58, 3.38)	1.92 (1.45, 3.19)	.449
Serum potassium, mmol/l	4.05 ± 0.29	3.96 ± 0.37	.231
Glycated hemoglobin, %	5.7 (5.5, 6.1)	5.7 (5.4, 6.1)	.559
Baseline medications, *n* (%)			
ACEIs/ARBs	38 (97.4%)	28(93.3%)	.407
GLP-1 receptor agonists	11 (28.2%)	3(10.0%)	.062
SGLT2 inhibitors	17 (43.6%)	17(56.7%)	.281
Statins	16 (41.0%)	14(46.7%)	.639
Diuretics	0	1(3.3%)	.895
Potassium-lowering agents	0	0	
Mean follow-up time, months	13.93 (13.07, 17.03)	14.62 (13.06, 17.77)	.541

Data are mean ± SD, medians [25th, 75th percentiles] or proportions. *P* values are obtained from Chi-squared test, Fisher's exact test, Mann–Whitney *U*-test, and Student's *t*-test as appropriate. A *P* value <.05 was considered significant.

There were no significant differences in the use of ACEIs/ARBs, SGLT2i, GLP-1R agonists, or statins between the two groups. The utilization of ACEIs/ARBs was 93.3% in the finerenone group and 97.4% in the control group. Two patients in the finerenone group were unable to tolerate ACEIs/ARBs therapy due to blood pressure intolerance, while one patient in the control group experienced similar intolerance (Table 1).

### Changes in proteinuria, eGFR, blood pressure, and serum potassium

**Figure 2:**
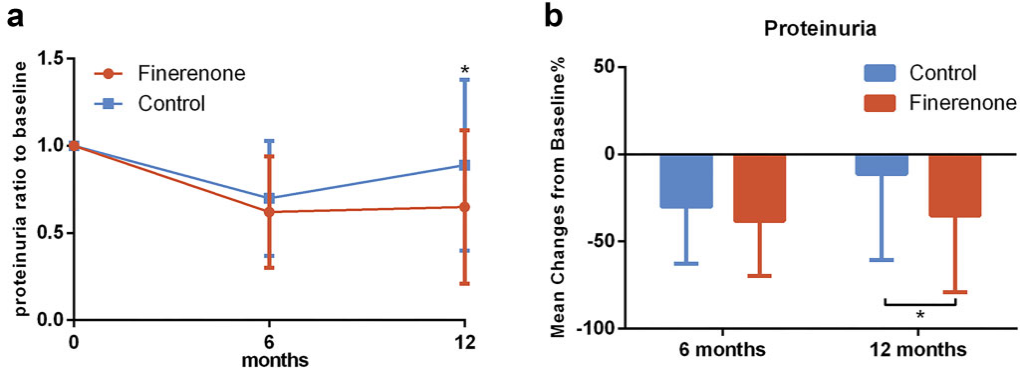
Change in proteinuria during follow-up. (**a**) Ratio of proteinuria to baseline. (**b**) Mean percentage change in proteinuria from baseline.

We evaluated the changes in 24-hour proteinuria, eGFR, blood pressure, and serum potassium at 6 months and 1 year of follow-up. Throughout the follow-up period, 24-hour proteinuria decreased in both groups; however, the percentage reduction in the finerenone group was significantly greater than that in the control group at 1 year of treatment (−35.03% vs. −11.20%, *P* = .010) (Fig. 2).

**Figure 3:**
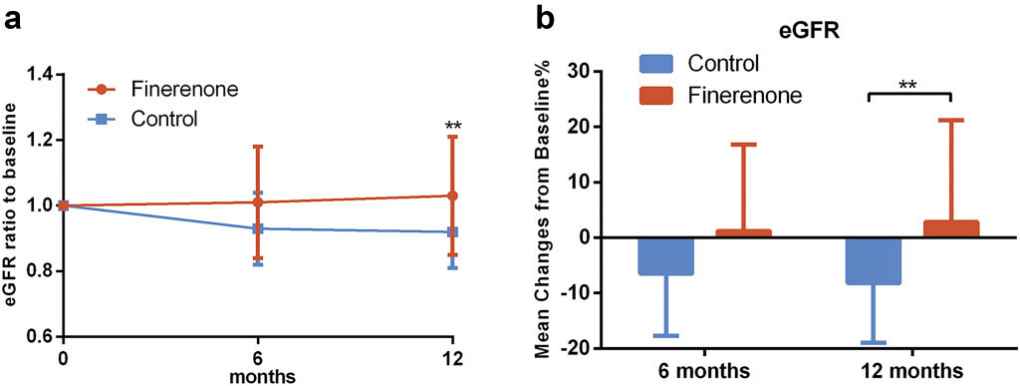
Change in eGFR during follow-up. (**a**) Ratio of eGFR to baseline. (**b**) Mean percentage change in eGFR from baseline.

For kidney function, the mean change in eGFR at 1 year of follow-up showed a significant difference between the finerenone group (2.85%) and the control group (−8.20%, *P* = .009) (Fig. 3). The mean absolute increase in eGFR at 1 year in the finerenone group was 1.1 ml/min per 1.73 m², while the mean absolute decrease in the control group was 7.26 ml/min per 1.73 m² (*P* = .001).

**Figure 4:**
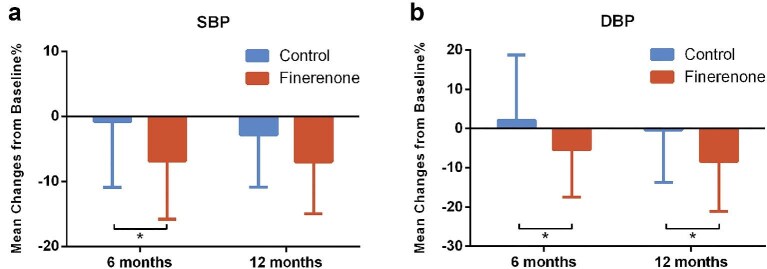
Change in blood pressure during follow-up. (**a**) Mean percentage change in SBP from baseline. (**b**) Mean percentage change in DBP from baseline.

Regarding blood pressure, finerenone treatment resulted in significantly greater reductions compared to controls (Fig. 4). For diastolic blood pressure (DBP), significant reductions were observed at both 6 months (5.40% reduction vs. 2.16% increase in controls, *P* = .025) and 12 months (8.35% vs. 0.34%, *P* = .046). Similarly, systolic blood pressure (SBP) showed a 6.82% reduction at 6 months (vs. 0.75%, *P* = .010), corresponding to an absolute decrease of 9.9 mmHg (vs. 1.97 mmHg, *P* = .013). This SBP reduction persisted at 12 months (10.07 vs. 4.44 mmHg, *P* = .045).

**Figure 5:**
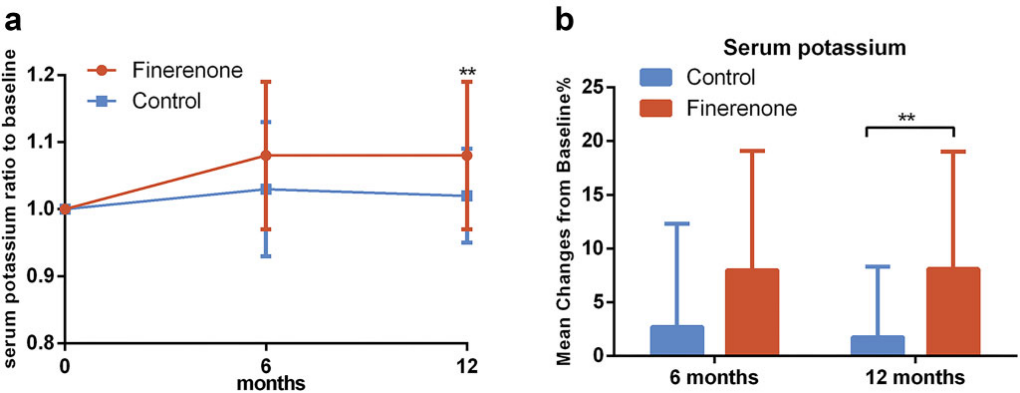
Change in serum potassium during follow-up. (**a**) Ratio of serum potassium to baseline. (**b**) Mean percentage change in serum potassium from baseline.

Finally, serum potassium levels increased in both groups during the follow-up period, with a significant increase in the finerenone group at 1 year of treatment compared to the control group (8.09% vs. 1.73%, *P* = .005) (Fig. 5).

### Side effects

No serious adverse events occurred during the follow-up period in either group (Table 2). Each group included one patient who developed acute kidney injury (AKI). Additionally, two cases of hyperkalemia were reported in the finerenone group, both occurring while patients were receiving a dose of 20 mg/day of finerenone. Following treatment for hyperkalemia, the finerenone dose was reduced to 10 mg/day, which successfully maintained serum potassium levels within the normal range in these two patients. Importantly, no severe hyperkalemia or adverse events requiring hospitalization due to hyperkalemia were observed in either group.

**Table 2:** Safety outcomes.

	Control	Finerenone	
Event	(*N* = 39)	(*N* = 30)	P values
Any adverse event	1	3	.190
Any serious adverse event	0	0	
Investigator-reported hyperkalemia	0	2	.102
Serious hyperkalemia	0	0	
Hospitalization due to hyperkalemia	0	0	
AKI	1	1	.850
Hospitalization due to AKI	0	0	
Cardiovascular events	0	0	

## DISCUSSION

This study is the first to evaluate the short-term efficacy and safety of finerenone in patients with ORG. The data demonstrate that patients receiving finerenone as an add-on to maximum tolerated RAS inhibitor therapy showed significantly reduced proteinuria, lower blood pressure and slower eGFR decline over 1 year compared with controls. Furthermore, finerenone was not associated with an increased incidence of adverse events.

The efficacy of finerenone in patients with T2DM and CKD is well-documented. The ARTS-DN study conducted in 2015 first demonstrated that finerenone reduces the UACR in patients with DN [9]. Subsequently, the FIDELIO-DKD and FIGARO-DKD trials further confirmed that finerenone significantly reduces the risk of CKD progression and cardiovascular events in patients with T2DM and CKD [10, 11]. Notably, subgroup analysis from the FIDELIO-DKD trial showed that the renal benefits of finerenone were even more pronounced in the Chinese population compared to the global cohort [13]. In addition, recent clinical observational studies have expanded the potential applications of finerenone to conditions such as Alport syndrome and non-diabetic CKD [14, 15]. However, its application in ORG has not been previously reported. Our study represents the first cohort analysis to evaluate the effect of short-term finerenone treatment on urinary protein levels and eGFR in patients with ORG. Most patients in both the finerenone and control groups were already receiving the maximum tolerated doses of ACEIs/ARBs, and the results suggest that the addition of finerenone to these therapies provides further renal benefits.

Our results indicate that in the control group, despite treatment with ACEIs/ARBs, the eGFR declined by 8.2% in the first year after treatment, with an average absolute decline of 7.26 ml/min/1.73 m². This decline is greater than anticipated. Previous cohort studies involving the Chinese population have demonstrated that 20.6% of patients with ORG had an eGFR decline slope of ≥5 ml/min/1.73 m^2^ per year during a median follow-up period of 45 months [16]. Furthermore, ∼10%–30% of ORG patients may ultimately progress to ESKD [3]. Notably, the FIDELITY study-a pre-specified pooled analysis of the FIDELIO-DKD and FIGARO-DKD trials-reported a chronic eGFR decline slope of −2.5 ml/min/1.73 m² per year in the finerenone group, compared to −3.7 ml/min/1.73 m^2^ per year in the control group [3]. In our study, eGFR remained stable in the finerenone group, with a slight increase of 1.1 ml/min/1.73 m^2^ at 1 year. These findings suggest that the decline in eGFR among ORG patients may not be a relatively slow or benign process, highlighting the significance of finerenone in mitigating eGFR decline. However, the long-term effects of finerenone on slowing eGFR decline in ORG patients require further investigation.

Furthermore, our study demonstrated significantly greater reductions in both SBP and DBP in the finerenone group compared with controls. These findings are consistent with existing evidence supporting finerenone's modest antihypertensive effects [10, 11, 17]. The observed blood pressure reductions may partially contribute to finerenone's renal protective effects in ORG, as suggested by the FIDELIO-DKD trial [18], where SBP reduction accounted for 13.8% of the primary kidney composite outcome benefit and 12.6% of the cardiovascular composite outcome benefit. Importantly, emerging mechanistic evidence indicates that the renoprotective effects of MRAs extend beyond blood pressure control, involving pleiotropic mechanisms including attenuation of oxidative stress, anti-inflammatory actions, and antifibrotic effects [19]. This suggests finerenone's cardiorenal protection likely involves both hemodynamic and non-hemodynamic mechanisms.

Our study also demonstrated the safety of finerenone in the treatment of ORG. The incidence of hyperkalemia in the finerenone group was 6.67%, with no cases necessitating treatment discontinuation or hospitalization due to hyperkalemia. By contrast, the control group reported no cases of hyperkalemia. In the FIDELITY study, the incidence of hyperkalemia was 14.01% in the finerenone group compared to 6.91% in the control group, with treatment discontinuation or hospitalization due to hyperkalemia occurring in 2.63% of the finerenone group versus 0.74% of the control group [20]. Notably, in our cohort, only four (10.3%) and three (10%) patients in the finerenone and control groups, respectively, were on double doses of ACEIs/ARBs. By contrast, the FIDELIO-DKD and FIGARO-DKD studies reported an ACEIs/ARBs usage rate at or above the maximum recommended dose of 42.34% in the finerenone group and 40.88% in the control group [10, 11]. The lower incidence of hyperkalemia observed in our study may be attributed to the relatively lower doses of ACEIs/ARBs and the shorter follow-up period.

The incidence of AKI in the finerenone group was 3.33%, compared to 2.56% in the control group, which aligns with the AKI incidence rates reported in the FIDELITY study: 3.38% in the finerenone group and 3.61% in the control group [20]. In our study, 56.7% of patients in the finerenone group and 43.6% in the control group were concurrently using SGLT2i. However, the incidence of AKI did not increase. Data from the FIDELITY study also indicated that the combination of finerenone and SGLT2i in patients with T2DM and CKD did not elevate the incidence of AKI, and instead, it reduced the incidence of hyperkalemia [21]. In 2023, a randomized controlled trial involving a non-diabetic CKD population demonstrated that the combination of finerenone and SGLT2i administered alongside the maximum tolerated dose of RAS inhibitors over a 4-week period, did not increase the incidence of AKI [15]. Additionally, a 2024 real-world study suggested that combining finerenone with SGLT2i in CKD patients led to a further reduction in UACR without increasing the rates of eGFR decline or hyperkalemia [22]. Nevertheless, the safety of triple therapy involving RAS inhibitors, SGLT2i, and MRAs requires further validation through large-scale prospective clinical trials.

The mechanisms underlying the efficacy of finerenone in treating ORG remain unclear. However, previous studies have indicated that multiple factors can activate MR in obese patients [23–25]. In obese animal models, MR inhibition has demonstrated renoprotective effects. For instance, eplerenone has been shown to improve glomerular hyperfiltration in obese canine models [26]. Furthermore, in hypertensive obese patients, the combination of the first-generation MRA spironolactone with RAS inhibitors, further reduced blood pressure and urinary albumin excretion rates [27]. However, the use of traditional steroidal MR antagonists in ORG is constrained by the risk of cross-reactivity with sex steroid receptors and the potential for hyperkalemia [28]. As a new-generation MRA, finerenone represents a promising therapeutic option for ORG.

This study has several limitations that should be considered. First, as a retrospective single-center study conducted in Han Chinese patients, the non-randomized design primarily allows for observational associations rather than definitive causal conclusions regarding finerenone's effects. Second, while we analyzed available data, missing follow-up measurements (including BMI, lipids, and glycated hemoglobin) may have influenced the efficacy assessments. Furthermore, the small size and the short follow-up period may limit the generalizability of our findings. Therefore, these limitations suggest that while our results are promising, they should be interpreted cautiously until confirmed by prospective multicenter studies with more diverse populations.

In conclusion, this study demonstrates that finerenone treatment in ORG patients is associated with significant reductions in proteinuria and blood pressure, along with attenuation of eGFR decline, while maintaining a favorable safety profile. However, further investigations are warranted to establish the long-term efficacy of finerenone in patients with ORG.

## Data Availability

The data underlying this article will be shared on reasonable request to the corresponding author.
